# Evaluation of ceftazidime/avibactam in combination with colistin against KPC-2-producing *Klebsiella pneumoniae* in static and dynamic time-kill experiments

**DOI:** 10.1093/jacamr/dlaf105

**Published:** 2025-06-18

**Authors:** Lisa Allander, Emma Vikdahl, Margarita Chatzopoulou, Amaury O'Jeanson, Linus Sandegren, Pernilla Lagerbäck, Thomas Tängdén

**Affiliations:** Department of Medical Sciences, Uppsala University, Uppsala, Sweden; Department of Medical Sciences, Uppsala University, Uppsala, Sweden; Department of Medical Sciences, Uppsala University, Uppsala, Sweden; Department of Pharmacy, Uppsala University, Uppsala, Sweden; Department of Medical Biochemistry and Microbiology, Uppsala University, Uppsala, Sweden; Department of Medical Sciences, Uppsala University, Uppsala, Sweden; Department of Medical Sciences, Uppsala University, Uppsala, Sweden

## Abstract

**Objectives:**

Ceftazidime/avibactam is used for severe infections caused by carbapenemase-producing *Klebsiella pneumoniae*. Combination therapy with older antibiotics is frequently used, but the supporting data are limited. This study aimed to evaluate ceftazidime/avibactam in combination with colistin against KPC-2-producing *K. pneumoniae*.

**Material and methods:**

Five clinical KPC-2-producing *K. pneumoniae* strains were characterized by phenotypic antibiotic susceptibility testing and whole-genome sequencing. Single antibiotics and combinations were evaluated in 24-h static time-kill experiments with ceftazidime/avibactam concentrations of 0.5× MIC_ratio_ and colistin at 0.5× and 1× MIC. One strain was subjected to 32-h dynamic time-kill experiments with ceftazidime/avibactam at concentrations mimicking patient pharmacokinetics in plasma and colistin added to 1 mg/L, i.e. the average free steady-state concentration. Population analysis was performed at 0, 16, and 32 h by plating at 4× and 8× MIC_ratio_ (ceftazidime/avibactam) or MIC (colistin) to assess resistance development.

**Results:**

All strains were susceptible to ceftazidime/avibactam and colistin, had mutations in *ompK35* and *ompK36*, and carried multiple β-lactamase genes. Ceftazidime/avibactam combined with colistin demonstrated 24-h synergy in static time-kill experiments against 3 of 5 strains. Although ceftazidime/avibactam and colistin alone showed rapid initial killing in the dynamic experiments, regrowth occurred after 4–8 h. The three-drug combination displayed a bactericidal effect and synergy at 14–32 h. Resistance development resulting in 64-fold MIC increases was observed in experiments with colistin alone.

**Conclusions:**

This study showed synergy with ceftazidime/avibactam and colistin against KPC-2-producing *K. pneumoniae* at clinically relevant concentrations. More studies are warranted to investigate the clinical potential of this combination.

## Introduction

Treatment options are limited for carbapenem-resistant *Enterobacterales* (CRE), which are typically multidrug resistant (MDR), and few novel antibiotics targeting these pathogens reach the market.^1^ Among CRE, *Klebsiella pneumoniae* is a predominant pathogen causing healthcare-associated infections, including pneumonia associated with high mortality in critically ill patients.^2,3^ A major mechanism of carbapenem resistance in *K. pneumoniae* is the production of carbapenemases, particularly KPC.^4^ In addition, clinical isolates often carry additional resistance mechanisms contributing to high-level resistance, e.g. co-production of β-lactamases, upregulation of efflux pumps, and reduction in drug influx through changes in outer membrane porins, such as OmpK35 and OmpK36.^4–6^

The global spread of KPC-producing *K. pneumoniae* has increased the reliance on last-resort antibiotics including the novel β-lactam/β-lactamase inhibitor combinations (BLBLIs). Ceftazidime/avibactam is currently recommended as a first-line option against CRE, except for MBL-producing strains.^7^ Avibactam inhibits AmpC β-lactamases, ESBLs (e.g. CTX-M-15), and serine carbapenemases (e.g. KPCs) but is inactive against metallo-β-lactamases (e.g. NDM).^8^ However, emergence of resistance to ceftazidime/avibactam during treatment has been reported, highlighting the need for alternative treatment strategies.^9,10^

Antibiotic combination therapy is used against CRE to enhance the activity of the available antibiotics.^11^ Although there are no clear recommendations for or against combination therapy, due to a lack of evidence, the new BLBLIs are frequently prescribed in combination with older antibiotics, such as polymyxins (polymyxin B and colistin).^7,11,12^ The polymyxins exhibit a bactericidal effect by destabilization of the bacterial outer membrane and can potentiate the activity of other antibiotics by increasing their entry into the cell, potentially resulting in synergy.^13^ This synergistic interaction may theoretically improve the effectiveness and delay emergence of resistance to ceftazidime/avibactam and other new last-resort BLBLIs.^14^

In this study, we assessed the activity of ceftazidime/avibactam and colistin against KPC-2-producing *K. pneumoniae* strains in 24-h static (five strains) and 32-h dynamic (one strain) time-kill experiments. The strains were characterized by MIC determination and whole-genome sequencing to identify genes and mutations that reduce susceptibility to β-lactams. Population analysis profiling was performed in the dynamic time-kill experiments to detect the emergence of resistance during antibiotic exposure.

## Materials and methods

*Bacteria, growth conditions, and antibiotics*: Five clinical KPC-2-producing *K. pneumoniae* were provided by the Public Health Agency of Sweden. The strains were collected between 2016 and 2019 as part of the Swedish national resistance surveillance programme and were isolated from clinical or screening cultures. Bacteria were grown at 37°C in cation-adjusted Mueller–Hinton (MH-II) (BD Diagnostics, Sparks, MD, USA) broth or on MH-II agar. Antibiotics were purchased from Sigma-Aldrich (Merck KGaA, Darmstadt, Germany) and dissolved according to the manufacturer’s instructions.

*Antibiotic susceptibility testing*: The susceptibility to standard antibiotics (amikacin, cefixime, cefotaxime, cefoxitin, ceftazidime, ceftibuten, ciprofloxacin, ertapenem, gentamicin, imipenem, meropenem, piperacillin/tazobactam, and trimethoprim/sulfamethoxazole) was assessed by disc diffusion performed at the accredited Clinical Microbiology Laboratory at the Uppsala University Hospital, Sweden, following routine procedures (ISO 15189). MIC determination for ceftazidime/avibactam and colistin was performed by broth microdilution according to EUCAST guidelines.^15–17^ EUCAST clinical breakpoints and epidemiological cut-off values (ECOFFs) were used for classification of susceptibility.^16,18,19^ In addition to the standard MIC determination for ceftazidime/avibactam (with the avibactam concentration fixed at 4 mg/L),^16^ we also determined the MIC with ceftazidime/avibactam at a ratio of 4:1, referred to as MIC_ratio_, which corresponds to the ratio administered to patients (2/0.5 g).^20^

*Genetic characterization*: Whole-genome sequencing was performed by the Public Health Agency of Sweden using IonTorrent S5 XL. *De novo* assembly with IonTorrent reads was performed in CLC Genomics Workbench 12.0 (CLCbio, Qiagen, Hilden, Germany) using the De Novo Assembly Pipeline v1.5. The strain used in the dynamic time-kill experiments (ARU871) was sequenced using both Illumina NovaSeq 6000 (Novogene, Cambridge, United Kingdom) and Oxford Nanopore GridION platform with R10.4 flow cells (Oxford Nanopore Technologies, Oxford, United Kingdom) to produce a complete genome sequence. Genomic DNA was prepared using the Epicentre MasterPure DNA purification Kit (Illumina Inc.) according to the manufacturer’s instructions. *De novo* assembly with Oxford Nanopore reads was performed in CLC Genomics Workbench 23 (CLC Bio, Qiagen, Hilden, Germany) using the De Novo Long Reads assembler v1.3 with the Long Read Support function v23.0. Polishing was done using the shortest subset of Nanopore reads, followed by a second round of polishing with Illumina reads to correct sequencing errors inherent in the Nanopore data.

The sequence type was established using MLST-2.0 version 2.0.9. Acquired resistance genes and chromosomal mutations known to affect antibiotic susceptibility were identified using ResFinder 4.5.0. Amino acid sequence variations in acquired resistance genes, porin genes (*ompK35*, *ompK36*, *ompK37*, *ompK26*, *lamB,* and *phoE*), the AcrAB-TolC efflux pump and its regulators (*acrA*, *acrB*, *acrR*, *tolC*, *ramR*, *ramA*, *marR*, *marA*, *marB*, *soxS, soxR,* and *robA*) were evaluated in CLC Main Workbench 24.0 (CLC bio, Qiagen, Hilden, Germany). Reference genes from the ResFinder database were used to identify amino acid sequence variations in acquired resistance genes. *K. pneumoniae* ATCC 35657 (NCBI Reference Sequence NZ_CP015134) was used as a reference for porin and efflux genes.

*Static time-kill experiments*: Bacterial cultures in the exponential phase were adjusted to starting inocula of ∼10^6^ cfu/mL. The activities of ceftazidime, avibactam, and colistin were evaluated alone and in two- or three-drug combinations. Ceftazidime/avibactam was tested at a concentration of 0.5× MIC_ratio_, and colistin was added to 0.5× and 1× MIC. Time-kill tubes were incubated at 37°C with shaking. Samples were taken at 0, 2, 4, 6, and 24 h, diluted in 10-fold dilution series, and 100 μL of diluted and undiluted samples were spread on agar plates and incubated at 37°C. Viable counts were read after 24 h. All experiments were performed in duplicates, and mean log_10_ cfu/mL values were used in the analysis.

*Antibiotic concentrations in the dynamic time-kill experiments*: The targeted pharmacokinetic (PK) profile of ceftazidime/avibactam was selected to mimic the free concentration in plasma of a typical pneumonia patient (age, 65 years; 70 kg, serum creatinine clearance (CrCL) of 80 mL/min) receiving 2/0.5 g q8h as 2-h infusions.^21^ The calculations assumed 10% and 8% plasma protein binding for ceftazidime and avibactam, respectively. Ceftazidime/avibactam was administered as a 2-h infusion from a media reservoir containing 108.6 mg/L ceftazidime and 23.1 mg/L avibactam. The flow rate was constant (52 mL/h) and was based on the working volume (100 mL) and the shortest half-life (avibactam, *t*_1/2_ = 1.33 h). During the 6-h elimination phase, the incoming medium contained 12.2 mg/L of ceftazidime to compensate for the longer half-life of this drug (*t*_1/2_ = 2.34 h). Colistin was injected as a loading dose of 2 mg/L to compensate for immediate surface binding.^22^ Additionally, the incoming medium was supplemented to contain 1 mg/L of colistin, corresponding to the average free steady-state concentration (*C*_ss_) in patient plasma.^13,23^

To verify that the desired PK profiles were achieved in the dynamic model, samples for drug concentration measurements were collected 20 min and 1, 2, 4, 6, 8, and 10 h after antibiotic administration in duplicate experiments without bacteria. For colistin, a 24-h sample was also collected, and all samples were added to an equal volume of 1% BSA (50/50, v/v) to avoid non-specific binding of colistin. A previously described agar diffusion assay was employed for ceftazidime/avibactam,^24^ using *E. coli* ATCC 25922 and *K. pneumoniae* ATCC 700603 as indicator organisms for ceftazidime and avibactam, respectively. When assessing avibactam concentrations, ceftazidime was added to a fixed concentration of 1 mg/L in the agar. Standard concentrations of 4, 8, 16, and 32 mg/L for ceftazidime and 2, 4, 8, and 16 mg/L for avibactam were used for the calibration curves. After 15–18 h of incubation at 37°C, the inhibition zones (mm) were measured. Concentrations were determined using a standard curve based on the linear relationship between zone diameter and the natural logarithm of the concentration. Colistin concentrations were measured using a liquid chromatography-tandem mass spectrometry (LC-MS/MS) method as previously described.^25^ The setup involved an Alliance 2695 LC system connected to a Quattro micro™ API triple quadrupole mass spectrometer (Waters SAS, Saint-Quentin-en-Yvelines, France). Calibration curves were prepared in a MH-II broth/BSA 1% solution (50/50, v/v), with concentrations ranging from 0.05 to 10 mg/L. Colistin was extracted through solid-phase extraction (SPE) using OASIS HLB cartridges (1 cc, Waters SAS, Saint-Quentin-en-Yvelines, France).

*Dynamic time-kill experiments*: A previously described *in vitro* PKPD model was used for the 32-h dynamic time-kill experiments.^24^ Bacteria in exponential growth phase were added to achieve a start inoculum of ∼10^6^ cfu/mL. Samples were collected for viable counts at 0, 1, and 2 h, then every second hour for 32 h. Samples were diluted in 10-fold dilution series, and 100 μL of diluted and undiluted samples was spread on agar plates and incubated at 37°C. Viable counts were read after 20–24 h. All experiments were performed in duplicates and mean log_10_ cfu/mL values were used in the analysis.

*Population analysis profiling*: Samples for population analysis profiling were collected from all dynamic experiments at 0, 16, and 32 h and stored at −80°C (10% DMSO). The samples were then thawed on ice and diluted in 10-fold dilution series. 100 μL of diluted and undiluted samples were spread on plates without antibiotics and on plates containing ceftazidime/avibactam at 4× and 8× MIC_ratio_ and colistin at 4× and 8× MIC. Bacterial growth was read after 24-h incubation at 37°C.

*Analysis of time-kill data*: No visible growth was recorded as the lower limit of detection (LOD, 1 log_10_ cfu/mL). A bactericidal effect was defined as ≥3 log_10_ reduction in cfu/mL compared to the starting inoculum. Synergy was defined as a ≥ 2 log_10_ decrease in cfu/mL with the combination compared to the most effective single treatment at the same time-point, and antagonism as a ≥ 2 log_10_ cfu/mL increase compared to either single antibiotic.^26^ The three-drug combinations were compared to the most effective single- or two-drug combination.

## Results

### Antibiotic susceptibility

All strains were multidrug resistant as determined with disk diffusion (Table S1, available as Supplementary data at *JAC-AMR* Online). Three strains (ARU871, ARU1019 and ARU922) were resistant to 12 of 13 tested antibiotics, ARU1011 was resistant to 11 antibiotics, and ARU1144 was resistant to 8 antibiotics. The strains were classified as susceptible to ceftazidime/avibactam (MICs, 1–8 mg/L) and colistin (MICs, 0.25–0.5 mg/L) according to MIC determination using the EUCAST clinical breakpoints (Table 1).^16^ The ceftazidime/avibactam MICs for ARU871 and ARU1019 did, however, exceed the ECOFF of 1 mg/L.^27^ The MIC_ratio_ values (i.e. susceptibility determined with ceftazidime/avibactam at a 4:1 ratio) were 2–4 times higher than the standard MICs (determined with 4 mg/L avibactam), suggesting a concentration-dependent effect of avibactam. For colistin, the EUCAST susceptibility breakpoint of 2 mg/L applies only for combination therapy with another active agent and is, in essence, the ECOFF.^19,28^

**Table 1.** Genetic characterization and antibiotic susceptibility (MIC and MIC_ratio_ values) for the tested *K. pneumoniae* strains

Strain	Sequence Type	Carbapenemase	Other β-lactamases	Major porins		MIC (mg/L)		
				OmpK35	OmpK36	CAZ/ AVI	CAZ/ AVI_ratio_	COL
ARU871	258	KPC-2	CTX-M-15 CMY-2 OXA-9 (W112*) OXA-10 TEM-1A TEM-1B SHV-12 SHV-182 (T102S)	E42fs	135_136insGD, 183_184_insLSP, GWS189_191TAL, F198Y, W207Y, H218N, TDE222_224LGD, VP228_229KL, S255T, 306delR, R347H	8 (S)	16:4	0.5 (S)
ARU1019	101	KPC-2	OXA-9 (W112*) TEM-1A SHV-28 (F3Y)	G62fs	136_137insDT, R188L	4 (S)	8:2	0.25 (S)
ARU922	258	KPC-2	OXA-9 (W112*) TEM-1A SHV-12	E42fs	135_136insGD, 183_184insLSP, GWS189_191TAL, F198Y, W207Y, H218N, TDE222_224LGD, VP228_229KL, S255T, 306delR, R347H	1 (S)	4:1	0.25 (S)
ARU1011	15	KPC-2	CTX-M-15 SHV-28		183_184insLSP, GWS189_191TAL, F198Y, W207Y, H218N, TDE222_224LGD, VP228_229KL, S255T, 306delR, R347H	1 (S)	4:1	0.25 (S)
ARU1144	11	KPC-2	CTX-M-15 OXA-1 TEM-1B SHV-182 (T102S)		183_184insLSP, GWS189_191TAL, F198Y, W207Y, H218N, TDE222_224LGD, VP228_229KL, S255T, 306delR, R347H	1 (S)	2:0.5	0.25 (S)

Amino acid changes in β-lactamases were compared to the reference sequence from ResFinder, while changes in the major porins (OmpK35/36) were compared to *K. pneumoniae* ATCC 35657 (NCBI NZ_CP015134). MICs were classified according to EUCAST clinical breakpoints, version 13.0.

CAZ, ceftazidime; CAZ/AVI, ceftazidime/avibactam with a constant avibactam concentration of 4 mg/L; CAZ/AVI_ratio_, ceftazidime/avibactam in ratio 4:1; COL, colistin; S, susceptible; R, resistant; fs, frameshift; *, premature stop codon; ins, insertion; del, deletion.

### Genetic characterization

Several β-lactamase genes were detected in addition to KPC-2, including CTX-M-15, CMY-2, OXA, TEM, and SHV-variants (Table 1). Moreover, we identified several amino acid sequence variations in genes encoding the major outer membrane porins OmpK35 and OmpK36. We identified loss of function mutations (frameshifts) in OmpK35 for three strains (ARU871, ARU1019, and ARU922). The frameshift mutation E42fs present in the ST258 strains ARU871 and ARU922 is a previously known truncation of OmpK35.^29^ All five strains had multiple amino acid variations in OmpK36, some of which are previously described.^30–32^ The biological significance of most variations is unclear, but the duplication of a glycine-aspartate (GD) pair (135_136InsGD) observed in OmpK36 of the two ST258 strains has been reported to reduce the entry of β-lactams.^30,33^ The GD insertion is located in a critical position of loop 3 and has been shown to narrow the porin channel. In contrast, the leucine–serine–proline (LSP) insertion in loop 4 (183_184insLSP) that we identified in OmpK36 in four of the tested strains has been reported to have no impact on pore reduction.^30^ No resistance determinants for colistin were found using the ResFinder tool.

Because of its use in the dynamic experiments, ARU871 underwent a more extensive genetic characterization. In this strain, we identified multiple genes associated with resistance against various antibiotics (Table S2), along with several amino acid sequence variations in chromosomal genes encoding porins, efflux pumps, and their regulators (Table S3). Inactivating changes were identified in OXA-9, Sul1 and RamA. However, the vast majority of the detected variations have unknown biological effects.

### Static time-kill experiments

**Figure 1.**
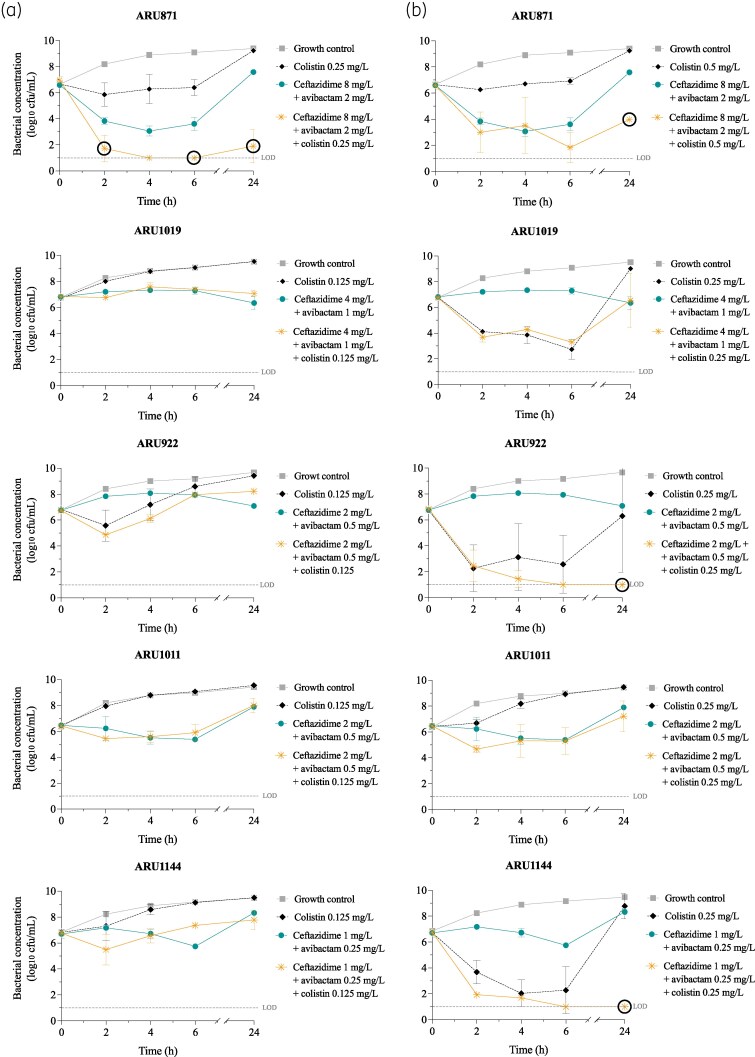
24-Hour static time-kill experiments with ceftazidime/avibactam at a concentration of 0.5× MIC_ratio_ and colistin at concentrations of (a) 0.5× MIC and (b) 1× MIC. Mean bacterial concentrations and standard deviations (error bars) are presented. Synergy is marked with a black circle. LOD, lower limit of detection.

Ceftazidime/avibactam at a concentration of 0.5× MIC_ratio_ and colistin at concentrations of 0.5× and 1× MIC were evaluated alone and in combination (Figure 1). Colistin alone at 0.5× MIC exhibited limited activity against all strains. An early bactericidal effect was observed with the higher concentration (1× MIC) at one or several time points (2–6 h) against ARU1019, ARU922, and ARU1144. However, regrowth always occurred within 24 h. Ceftazidime/avibactam alone was only bactericidal against ARU871 at 4 and 6 h. The three-drug combination of ceftazidime/avibactam and colistin at 1× MIC demonstrated synergy at 24 h against three of five strains (ARU871, ARU922, and ARU1144). When using the lower colistin concentrations of 0.5× MIC, synergy was only found against ARU871 (at 2, 6, and 24 h). No synergistic effect was observed against ARU1019 or ARU1011.

### Dynamic time-kill experiments

ARU871 was selected for the dynamic experiments because it had the highest MICs. This strain was consequently considered the most difficult-to-treat pathogen and to represent a situation where combination therapy was most likely to be beneficial. Moreover, the static experiments demonstrated synergy against this strain at both colistin concentrations. Drug concentration measurements showed acceptable agreement between the desired and observed PK profiles for ceftazidime (C_max_ 67.03 mg/L, SD 0; C_min_ 11.32 mg/L, SD 0.59) and avibactam (C_max_ 13.72 mg/L, SD 1.24; C_min_, below the LOD of 2 mg/L) (Figure S1). The measured mean colistin concentration was 0.49 mg/L (SD 0.09 mg/L) (Table S4), i.e. ca. 50% lower than the concentration added to the medium (1 mg/L), likely due to non-specific surface binding.^22^

**Figure 2.**
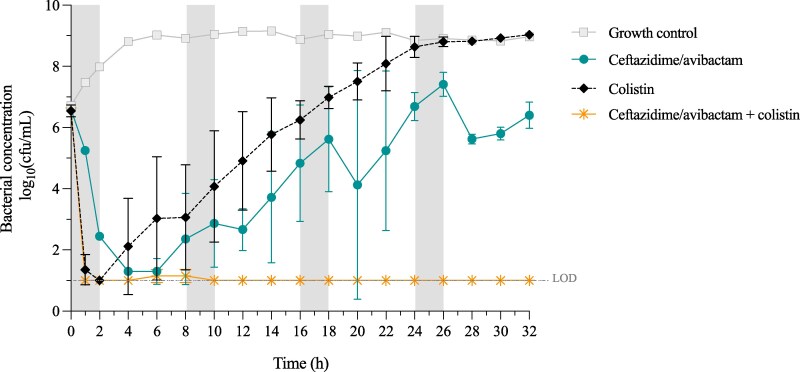
Dynamic time-kill experiments with ceftazidime/avibactam and colistin against *K. pneumoniae* ARU871. Mean bacterial concentrations and standard deviations (error bars) are presented. The 2-hour infusion times with ceftazidime/avibactam are denoted in grey. LOD, lower limit of detection.

Ceftazidime/avibactam caused considerable initial killing; bacterial concentrations were <1.5 log_10_ cfu/mL within 4 h after the first dose, and the effect was bactericidal between 2 and 12 h (Figure 2). However, regrowth was apparent already from 8 h, and the three consecutive doses had lesser effects on the bacteria. Colistin alone also caused rapid killing, resulting in bacterial concentrations below LOD (1 log_10_ cfu/mL) at 2 h and a sustained bactericidal effect up to 8 h. Following regrowth, however, the bacterial concentration exceeded the start inoculum from 18 h and onwards. The three-drug combination resulted in a rapid and substantial decrease in bacterial concentrations. A sustained bactericidal effect was demonstrated within 1 h and throughout the 32-h experiment. Bacterial growth was undetectable at all time points except at 6 and 8 h. Consequently, the combination was classified as synergistic at all time points between 14 and 32 h (Figure 2).

### Population analysis profiling

**Figure 3.**
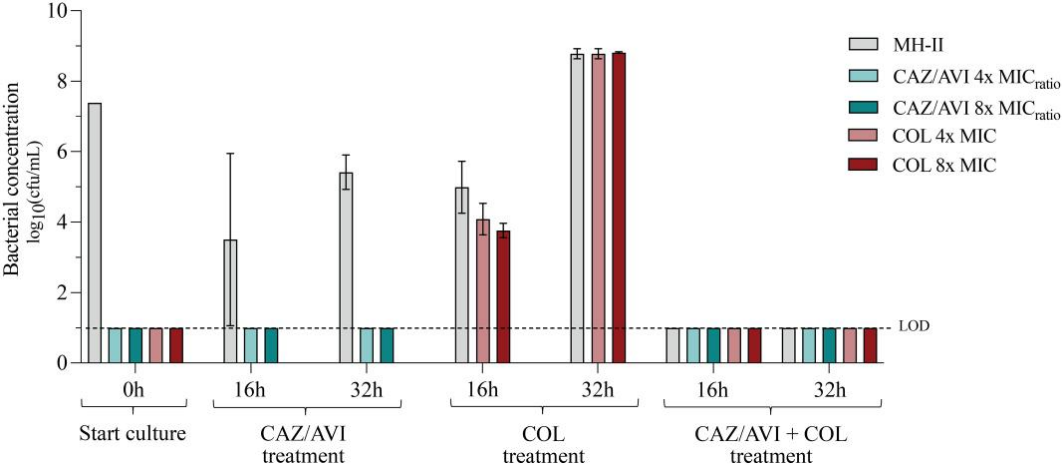
Population analysis profiling at 0, 16, and 32 h from the dynamic time-kill experiments with ARU871. Mean values and standard deviations (error bars) of viable counts on non-selective plates (MH-II), 4× and 8× MIC_ratio_ ceftazidime/avibactam and 4× and 8× MIC colistin are presented. MH-II, cation-adjusted Mueller–Hinton; CAZ/AVI, ceftazidime/avibactam; COL, colistin; LOD, lower limit of detection.

Population analysis profiling was performed to assess the emergence of resistance in the dynamic time-kill experiments (Figure 3). No bacterial growth was observed on antibiotic-containing plates prior to drug exposure or after exposure to ceftazidime/avibactam alone or the three-drug combination. During exposure to colistin alone, resistant colonies appeared in the populations at 16 and 32 h in both replicates. MIC testing of six isolated colonies each (three from each replicate) from 4× and 8× MIC plates revealed a 64-fold increase in colistin MIC (32 mg/L) for all resistant isolates.

## Discussion

This study reports an improved antibacterial activity of ceftazidime/avibactam when combined with colistin against multidrug-resistant KPC-2-producing *K. pneumoniae* strains. According to standard MIC determination, the tested strains were phenotypically susceptible to both ceftazidime/avibactam and colistin. Yet, the three-drug combination demonstrated synergy against three of five strains in static time-kill experiments at clinically achievable concentrations. Moreover, bactericidal and synergistic effects were observed against the least susceptible strain in dynamic experiments with ceftazidime/avibactam at concentrations mimicking patient plasma PK and colistin at a low concentration below the average *C*_ss_.

Genetic characterization of the strains revealed that several β-lactamases, including ESBLs, were present in addition to the KPC-2 enzyme. All detected β-lactamases are inhibited by avibactam. Nevertheless, the co-production of multiple enzymes or increased gene expression can lead to a reduction in susceptibility to BLBLIs.^4^ While β-lactamases are the main drivers of high-level resistance, reduced membrane permeability also contributes to resistance against β-lactams and BLBLIs.^6,29^ Modifications of OmpK35 and OmpK36 in conjunction with other resistance mechanisms have frequently been associated with reduced susceptibility to new BLBLIs, including ceftazidime/avibactam.^29,34^ Amino acid sequence variations in these porins were common in our study: OmpK35 was truncated in three strains, and all five strains had multiple amino acid variations in OmpK36. Some variations were consistent both among and across different sequence types, potentially attributable to clonal relationships or variations in the reference sequence.

Mechanistically, colistin may counteract reduced outer membrane permeability by membrane destabilization and disruption. In the static time-kill experiments, synergy with ceftazidime/avibactam and colistin was observed in at least one time-point against the two ST258 strains with the OmpK35 truncation (E42fs) and GD duplication in OmpK36 (ARU871 and ARU922), which are commonly reported in ST258 and known to reduce entry of β-lactams into the cell.^29,32^ However, synergy was also detected against one strain without known membrane permeability alterations (ARU1144). In line with our finding, a previous study investigating the influence of *ompK36* genotypes on combination effects in time-kill experiments, reported synergy with ceftazidime/avibactam and colistin independent of the GD insertion in OmpK36.^35^

Regrowth was observed following exposure to ceftazidime/avibactam alone and colistin alone in the dynamic experiments and across strains in the static time-kill experiments. In the static time-kill experiments, regrowth was also observed in two strains following treatment with ceftazidime/avibactam and colistin in combination. In the dynamic experiments, the emergence of resistance to colistin was noted already after 16 h of single-drug exposure. In contrast, despite marked regrowth between doses and reduced killing effect at later doses, no resistance development was detected during exposure to ceftazidime/avibactam alone. This finding aligns with a study testing a hypervirulent KPC-2-producing *K. pneumoniae* (MIC 4 mg/L) in a dynamic model where no MIC increases were detected in spite of regrowth after exposure to ceftazidime/avibactam.^36^ In that study, transcriptomic analyses suggested that perturbations to cell respiration, carbohydrate transport, and stress-response contributed to the antibiotic tolerance. Emergence of resistance was not evaluated in our static time-kill experiments but could be a reason for the regrowth observed across the strains. Additionally, regrowth may also be attributed to antibiotic degradation during experiments, as well as drug depletion due to binding to the target and the combined activity of multiple β-lactamases.

To our knowledge, no previous dynamic time-kill studies have evaluated the combination effect of ceftazidime/avibactam and polymyxins against KPC-producing *K. pneumoniae.* Static time-kill experiments have yielded inconsistent findings.^37–43^ Similarly, some *in vivo* studies reported improved effects with polymyxin combinations,^37,42^ whereas others found no enhanced activity compared to ceftazidime/avibactam alone.^38^ The variability in results may be due to methodological differences and strain-dependent effects. For example, co-production of other β-lactamases, levels of membrane permeability, or efflux may significantly impact the susceptibility to the single drugs and the combination.

Differences in drug concentrations are also likely to have caused variability in results across studies. In most cases, ceftazidime/avibactam was tested using a constant avibactam concentration (4 mg/L). While this approach is recommended for MIC determination,^16,44^ it does not reflect the doses used for administration to patients (2/0.5 g). Using a fixed concentration of avibactam may thus lead to under- or overestimation of its clinical efficacy, depending on the site of infection and the susceptibility of the pathogen. In this study, we evaluated the activity of ceftazidime/avibactam at a 4:1 concentration ratio. Although there were no major differences (2–4-fold) between MIC and MIC_ratio_, the avibactam concentrations were invariably <4 mg/L (0.25–2 mg/L) in the static experiments, which may have resulted in a lower probability of detecting synergy compared to other studies.

Strengths of this study include the genetic characterization, the simulation of patient pharmacokinetics of ceftazidime/avibactam and frequent sampling for viable counts conducted during the dynamic time-kill experiments. We observed some variability in bacterial killing and growth dynamics between replicates of the time-kill experiments, which is expected and may partly be attributed to biological variation. A limitation of the study is that it only included five strains. Because the antibacterial effects of single antibiotics and the combinations may be strain-dependent, the results cannot be generalized to all KPC-producing *K. pneumoniae*. The low number of strains also prevents conclusions on associations between bacterial genetics and synergy. Yet, the study offers important directions for future research to validate our findings. *In vitro* findings cannot be directly translated to the clinical setting for several reasons, including differences in growth conditions and the lack of immune system effects. Nephrotoxicity is an important concern when using colistin in clinical practice.^13^ However, this side effect is usually reversible and may be outweighed by the benefits of combination therapy if evidence supports an enhanced therapeutic efficacy against severe infections and suppression of resistance development.

In conclusion, the combination of ceftazidime/avibactam and colistin showed synergistic potential against clinical KPC-2-producing *K. pneumoniae* in static and dynamic *in vitro* time-kill experiments. More research is warranted to validate our findings, determine the clinical value of this combination, and assess the mechanisms of synergy.

## Supplementary Material

dlaf105_Supplementary_Data

## Data Availability

Raw data from sequencing are deposited in the NCBI Sequence Read Archive under BioProject accession number PRJNA892093 and PRJNA1261832.

## Supplementary data

Figure S1 and Tables S1 to S4 are available as Supplementary data at *JAC-AMR* Online.
