# Supplementary material for: Evaluation of ceftazidime/avibactam in combination with colistin against KPC-2-producing Klebsiella pneumoniae in static and dynamic time-kill experiments
Source: JAC Antimicrob Resist. 2025 Jun 18;7(3):dlaf105. doi: 10.1093/jacamr/dlaf105 (PMC12203663; doi:10.1093/jacamr/dlaf105)
Supplement: dlaf105_Supplementary_Data [file dlaf105_supplementary_data.pdf]

Supplementary Material

Evaluation of ceftazidime/avibactam in combination with colistin against KPC-2-producing *Klebsiella pneumoniae* in static and dynamic time-kill experiments

Lisa ALLANDER[1], Emma VIKDAHL[1], Margarita CHATZOPOULOU [1], Amaury O'JEANSON [2], Linus SANDEGREN [3], Pernilla LAGERBÄCK[1], Thomas TÄNGDÉN[1]

[1] Department of Medical Sciences, Uppsala University, Uppsala, Sweden.

[2] Department of Pharmacy, Uppsala University, Uppsala, Sweden.

[3] Department of Medical Biochemistry and Microbiology, Uppsala University, Uppsala, Sweden.

Table S1. Antibiotic susceptibility according to disc diffusion.

**Antibiotic**	**Strain**				
	ARU871	ARU1019	ARU922	ARU1011	ARU1144
Amikacin	R	R	R	S	S
Cefixime	R	R	R	R	R
Cefotaxime	R	R	R	R	R
Cefoxitin	R	R	R	R	S
Ceftazidime	R	R	R	R	R
Ceftibuten	R	R	R	R	R
Ciprofloxacin	R	R	R	R	R
Ertapenem	R	R	R	R	R
Gentamicin	S	R	S	S	S
Imipenem	R	R	R	R	S
Meropenem	R	R	R	R	S
Piperacillin-tazobactam	R	R	R	R	R
Trimethoprim-sulfamethoxazole	R	S	R	R	R

Abbreviations: S, susceptible; R, resistant.

Table S2. Resistance genes identified in ARU871 using ResFinder version 4.5.0 (Center for Genomic Epidemiology). Amino acid variations are assessed in relation to the reference sequence provided by ResFinder. No resistance genes for colistin were detected. Variations in porin-encoding genes and AcrAB-TolC efflux genes are excluded and can be found in table S2.

**Resistance gene**			**Amino acid variation**
Aminoglycoside-modifying enzymes	Aminoglycoside 3'-phosphotransferase	*aph(3')-Ia*	
	Aminoglycoside nucleotidyltransferase	*aadA1*	A4V
		aadA2	
	Aminoglycoside acetyltransferase	*aac(6')-Ib3*	N5T
		aac(6')-Ib	
β-lactamases	AmpC cephalosporinases	$bla_{\text{CMY-2}}$	
	Carbapenemase	$bla_{\text{KPC-2}}$	
	ESBLs	$bla_{\text{CTX-M-15}}$	
		$bla_{\text{SHV-12}}$	
	Other β-lactamases	$bla_{\text{OXA-9}}$	W112*
		$bla_{\text{OXA-10}}$	
		$bla_{\text{SHV-182}}$	T102S
		$bla_{\text{TEM-1A}}$	
		$bla_{\text{TEM-1B}}$	
Chloramphenicol acetyltransferase		*catA1*	
DNA gyrase		*gyrB*	E466D
Glutathione S-transferase		*fosA*[1]	I91V, Q130P, D138E
Macrolide phosphotransferase		*mph(A)*	
		mph(B)	
Major facilitator superfamily (MFS) antibiotic efflux pump		*cmlA1*	S2R, L304S, A328S, G412E
		floR	I349V, T363A, A389V, S393L, D395G
Quinolone resistance protein (qnr)		*qnrA1*	
Resistance-nodulation-cell division (RND) efflux pump		*OqxA*	
		Oqx[1]	
Sulfonamide-resistant dihydropteroate synthase		*sul1*	99Yfs
		sul1	
		sul2	
Trimethoprim-resistant dihydrofolate reductase		*dfrA12*	

Abbreviations: fs, frameshift; *, premature stop codon.

Table S3. Amino acid sequence variations in ARU871 chromosomal genes encoding porins, efflux pumps and their regulators. *K. pneumoniae* ATCC 35657 (NCBI NZ_CP015134) was used as the reference sequence.

	**Gene**	**Amino acid variation**
Porins	*ompK35*	E42fs, G109*
	ompK36	135_136InsGD, 183_184_InsLSP, G189T, W190A, S191L, F198Y, W207Y, H218N, T222L, D223G, E224D, V228K, P229L, S255T, 306DelR, R347H
	ompK37	N230G, M233Q, 233_234InsHYTH, Q235E, T236R, N237Y, R239K, E244D, N274S, D275T, 275_276InsSSTNGG, V277I
	ompK26	L94F
	lamB	N171K
	phoE	
AcrAB-TolC efflux genes	*robA*	
	acrA	A188T[1]
	acrB	R23G
	acrR	
	tolC	
	marR	I11M, E85K
	marA	
	marB	
	soxS	
	soxR	
	ramR	61_62InsLA[2]
	ramA	┼

Abbreviations: fs, frameshift; *, premature stop codon; ┼, truncated gene (due to a ~4 kb deletion in the region); Ins, insertion; Del, deletion.

[1] Previously reported and predicted as a neutral mutation (Li et al. 2023, PMID: 36847569).

[2] The insertion results in the absence of a stop codon compared to the annotated gene in the reference sequence. Including three downstream nucleotides introduces a stop codon consistent with other *ramR* variants, as confirmed by Uniprot references (CP003200, HTHTR_KLEPH-RamR).

Table S4. Assessment of colistin concentrations in the PKPD model (LC-MS/MS). Mean concentrations and standard deviations (SD) are presented.

**Time after dosing**	**Colistin concentration (mg/L)**		
	Replicate 1	Replicate 2	± SD
15 min	0.73	0.53	0.14
1 h	0.54	0.56	0.02
2 h	0.47	0.48	0.01
4 h	0.42	0.48	0.04
6 h	0.47	0.39	0.06
8 h	0.41	0.36	0.03
10 h	0.39	0.50	0.08
24 h	0.52	0.53	0.01
Average concentration: 0.49 ± 0.09			

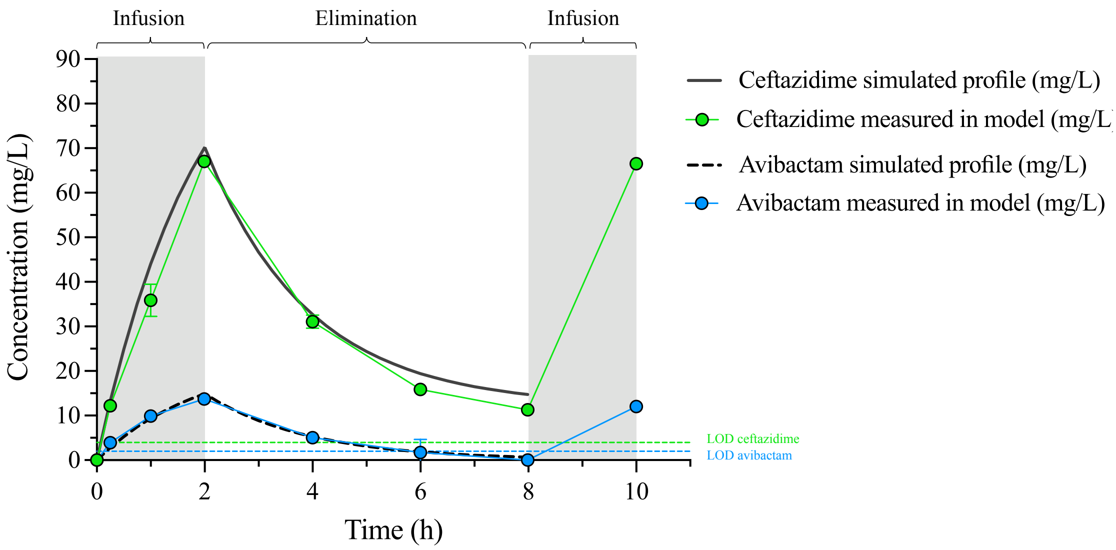

Abbreviations: LOD, lower limit of detection.

Figure S1. Assessment of ceftazidime and avibactam concentrations in the PKPD model. Mean concentrations and standard deviations (error bars) are presented. The 2-hour infusion times are denoted in grey. Green and blue dashed lines indicate the lower limit of detection for ceftazidime (4 mg/L) and avibactam (2 mg/L), respectively.
